# Genomic Regions and Candidate Genes Associated with Milk Production Traits in Holstein and Its Crossbred Cattle: A Review

**DOI:** 10.1155/2023/8497453

**Published:** 2023-07-27

**Authors:** R. Bekele, M. Taye, G. Abebe, S. Meseret

**Affiliations:** ^1^School of Animal and Range Sciences, College of Agriculture, Hawassa University, P.O. Box 5, Hawassa, Ethiopia; ^2^Department of Animal Science, College of Agriculture and Natural Resource Sciences, Debre Berhan University, P.O. Box 445, Debre Berhan, Ethiopia; ^3^Livestock Genetics, International Livestock Research Institute (ILRI), Addis Ababa, Ethiopia

## Abstract

Genome-wide association studies (GWAS) are a powerful tool for identifying genomic regions and causative genes associated with economically important traits in dairy cattle, particularly complex traits, such as milk production. This is possible due to advances in next-generation sequencing technology. This review summarized information on identified candidate genes and genomic regions associated with milk production traits in Holstein and its crossbreds from various regions of the world. Milk production traits are important in dairy cattle breeding programs because of their direct economic impact on the industry and their close relationship with nutritional requirements. GWAS has been used in a large number of studies to identify genomic regions and candidate genes associated with milk production traits in dairy cattle. Many genomic regions and candidate genes have already been identified in Holstein and its crossbreds. Genes and single nucleotide polymorphisms (SNPs) that significantly affect milk yield (MY) were found in all autosomal chromosomes except chromosomes 27 and 29. Half of the reported SNPs associated with fat yield and fat percentage were found on chromosome 14. However, a large number of significant SNPs for protein yield (PY) and protein percentage were found on chromosomes 1, 5, and 20. Approximately 155 SNPs with significant influence on multiple milk production traits have been identified. Several promising candidate genes, including diacylglycerol O-acyltransferase 1, plectin, Rho GTPase activating protein 39, protein phosphatase 1 regulatory subunit 16A, and sphingomyelin phosphodiesterase 5 were found to have pleiotropic effects on all five milk production traits. Thus, to improve milk production traits it is of practical relevance to focus on significant SNPs and pleiotropic genes frequently found to affect multiple milk production traits.

## 1. Introduction

Milk is a highly nutritious and valuable human food consumed by millions of people every day in a variety of flavors and products. Milk production traits, such as milk, fat, and protein yields (PYs), and fat and protein percentages (PPs), are the essential economic traits that are used to evaluate milk quantity and quality and play a major role in dairy development [1]. Milk traits are influenced by multiple genes, and therefore genomic evaluations have the potential to rapidly increase the rate of genetic improvement for these traits in dairy [2]. Understanding genetic variation in dairy cattle is crucial to associating genomic regions with milk yield (MY) and composition traits. The sequencing of the bovine genome in 2004 sparked a worldwide effort to improve how cattle genetic values can be estimated using basic genetic coding information [3].

Detecting genomic regions will help to identify potential candidate genes that may be responsible for genetic variation in MY and milk composition traits. These candidate genes could help to improve our understanding of the biological background of milk production traits. Genome-Wide Association Studies (GWAS) are a popular method for determining, which genes and gene regions influence the expression of specific phenotypes by identifying single nucleotide polymorphisms (SNPs) associated with the phenotypes across the whole genome [4, 5]. GWAS can effectively identify potential genetic variants associated with quantitative traits, and facilitate the utilization of molecular information for genomic selection in dairy cattle [6, 7].

GWAS have been extensively used in recent years to identify genomic regions and candidate genes for milk production traits in Holstein and its crossbreds in cattle populations from various countries. Numerous candidate genes and quantitative regions associated with milk production traits in Holstein and its crossbreds have already been identified [7–9, 16, 17]. The objective of this review was to summarize the findings of genomic regions and candidate genes associated with milk production traits including MY, FY, PY, FP, and PP in Holstein and its crossbreds.

## 2. Methods

Data were gathered from Google Scholar, Science Direct, PubMed, Springer Link, Web of Science, and Scopus using the keywords GWAS, genomic markers, Holstein, crossbred, and milk production traits. The current review included published studies that discussed candidate genes and genomic regions that were significantly associated with milk production traits in Holstein and its crossbreds. We included studies that used a *P*-value as a statistical significance criterion. In addition, we included studies that reported both SNPs and candidate genes. Similarly, only articles published in English in peer-reviewed journals since 2009 were included in this review. Thus, conference papers, books, book chapters, theses, and unpublished results were excluded from this review. To ensure consistency throughout the review, SNP names that differed from what researchers reported were converted to the rs name format.

## 3. GWAS for Milk Production Traits in Holstein and Its Crossbreds

The phenotypic expression of milk production traits (MY and milk composition) is controlled by many genes. The detection of potential candidate genes affecting milk production traits of cattle is made possible by the widespread availability of SNP markers through the fast-growing number of genotyped cattle [16]. Several GWAS focused on the identification of potential candidate genes and genomic regions underlying milk production traits (MY, FY, FP, PY, and PP). Most researchers conducted association studies using 50 K chips, except [7, 18]; who used 26 and 100 K chips, respectively. The methodologies they used were linear, single-locus, multi-locus, and Bayesian mixed models. This review summarized the 462 significantly associated SNPs from which 34 SNPs for milk production traits were repeatedly reported by various researchers at different rates. Ten SNPs were reported three and more than three from 34 SNPs: rs109421300, rs109350371, rs109146371, rs109558046, rs109752439, rs109234250, rs109968515, rs110199901, rs17870736, and rs43703011. While the ramming 24 SNPs were reported twice. For instance, rs109421300 was reported by [11, 13, 14, 17, 21].

Diacylglycerol O-acyltransferase 1 (DGAT1) was the most frequently reported candidate gene associated with one or more milk production traits by multiple authors [9, 11, 13, 14, 17, 19, 22]. GHR was reported by [11, 13, 21, 23]. MAPK15 was reported by [15, 21, 24]. KHDRBS3 was reported by [7, 16, 21]. The remaining candidate genes were reported by fewer than four researchers. Researchers [8, 16, 18] conducted association studies for milk production traits with crossbred dairy cattle ranging from 87.50% to <100% Holstein, Holsteinized Black-and-White Pied and Gir × Holstein (Girolando) in Thailand, Russia, and Brazil, respectively, using a single marker linear model. The remaining studies included in this review were conducted with Holstein and its crossbreds.

### 3.1. Milk Yield

**Figure 1:**
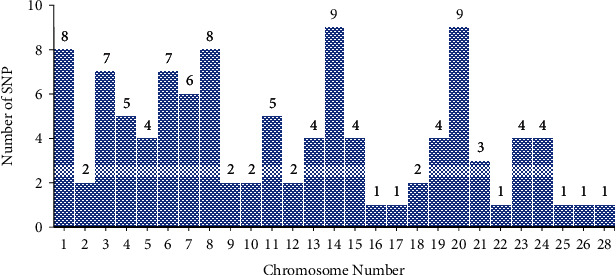
The number of significant SNPs associated with MY found in chromosomes from Holstein and its crossbreds.

MY is the most economically important trait, and several researchers were keenly interested in identifying the genes and genomic regions that contribute to its variation in Holstein and its crossbreds [7, 11, 13, 16, 17]. Several publications that utilized GWAS for the MY are shown in Table 1. These researchers reported 103 individual SNPs that were significantly associated with MY. These SNPs were found on all autosomal chromosomes except chromosomes 27 and 29 in Holsteins and their crossbreds.  Figure 1 shows the frequency of SNPs identified by different researchers within each chromosome. Chromosomes 14 and 20 have a high number of SNPs. This information could be used to help focus research on these two chromosomes to improve MY.

The candidate genes significantly affecting MY that were reported more than twice (Table 1) were GNA14 in Thai Holstein crossbreds [8], PTBP2 in U.S. Holstein [19], and U6 in Brazilian Holstein crossbreds [16].

### 3.2. Fat Yield and Fat Percentage

**Figure 2:**
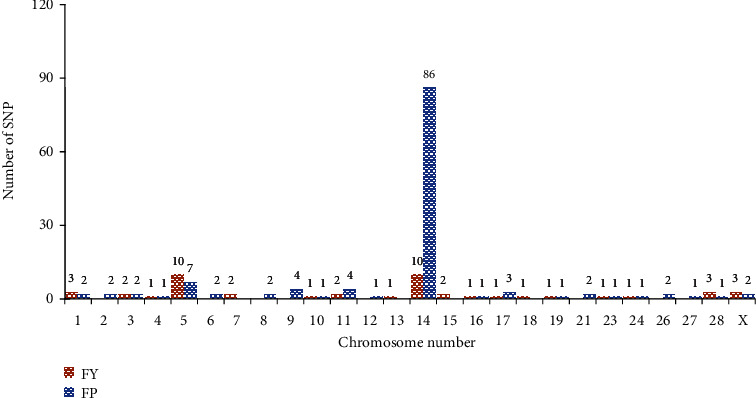
The number of significant SNPs associated with FY and fat percentage (FP) in chromosomes from Holstein and its crossbreds.

Fat is an important component of milk and it is controlled by gene networks associated with several metabolic and biological pathways. The identification of potential genes and their locations can provide valuable information that can be used for selective breeding to improve milk quality. A total of 46 significantly associated SNPs with FY and 117 significantly associated SNPs with FP were detected in various chromosomes from Holstein and its crossbreds. Several researchers [9, 12, 17, 19, 20] mentioned more than twice that two SNPs (rs109350371 and rs109421300) that were significantly associated with FP.  Figure 2 shows the number of identified significant SNPs associated with FY and FP in chromosomes from Holstein and its crossbreds. Chromosome 14 contains a large number of significant SNPs associated with FP accounting for more than 75% of the SNPs on this chromosome. Conversely, for fat yield (FY), chromosomes 5 and 14 have an equal number of significantly associated SNPs.

A detailed list of the candidate genes, significant SNPs, and chromosome numbers for FY and FP is presented in Table 2. Several candidate genes influence the expression of FY, including inositol 1,4,5-trisphosphate receptor, type 2 (ITPR2), ATP-binding cassette sub-family C member 9 (ABCC9), sulfonylurea receptor 2 (SUR2), cleavage and polyadenylation specific factor 1 (CPSF1), DGAT1, phosphodiesterase 4B (PDE4), and methyl transferase like 15 (METTL15) reported by [7, 12, 13, 17, 25]. Similarly, multiple candidate genes influence the expression of FP, including 5-oxoprolinase, ATP-Hydrolysing (OPLAH), G protein-coupled receptor 20 (GPR20), collagen type XXII alpha 1 chain (COL22A1), glutamate receptor ionotropic NMDA type subunit associated protein (GRINA), forkhead box H1 (FOXH1), microsomal glutathione S-transferase 1 (MGST1), ephrin type-receptor A6 (EPHA6), and alanine and arginine rich domain containing protein (AARD) reported by [9, 11, 13, 15, 19, 21, 27].

### 3.3. Protein Yield and PP

**Figure 3:**
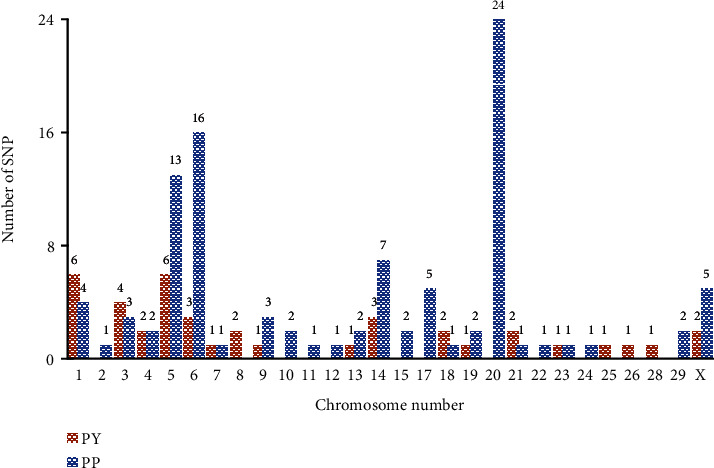
The number of significant SNPs associated with PY and protein in chromosomes from Holstein and its crossbreds.

Candidate genes, significant SNPs, and chromosome numbers for PY and PP are presented in Table 3. There were 44 significantly associated SNPs for PY and 101 significantly associated SNPs for PP in Holstein and its crossbreds.  Figure 3 shows the number of significant SNPs associated with PY and PP in chromosomes from Holstein and its crossbreds. Many significant SNPs were reported on chromosome 20, and about half of the significant SNPs for PP were identified on chromosomes 20, 6, and 5. In addition, chromosomes 1 and 5 had a large number of significant SNPs for PY.

Table 3 shows potential genes, significant SNPs, and chromosomes associated with PY and PP. Genes associated with PY, included pyruvate dehydrogenase E1 subunit alpha 2 (PDHA2), C-terminal binding protein 2 (CTBP2), mitogen-activated protein kinase 9 (MAPK9), Hermansky-Pudlak syndrome-3 (HPS3), ADP ribosylation factor guanine nucleotide exchange factor 1 (ARFGEF), solute carrier organic anion transporter family member 1A2 (SLCO1A2), major facilitator superfamily domain containing 1 (MFSD1) [7, 14, 20, 21, 24, 25]. Findings indicate several potential genes associated with PP, for example, growth hormone receptor (GHR), nipped-B-like protein (NIPBL), platelet-derived growth factor receptor alpha (PDGFRA), peroxisome proliferator-activated receptor gamma coactivator 1-alpha (PPARGC1A), casein kappa (CSN3), RNA polymerase II associated protein 3 (RPAP3), solute carrier family 1 member 3 (SLC1A3), and zinc finger protein 384 (ZNF384) [7, 11, 14, 19, 22, 23].

### 3.4. All Milk Production Traits

**Figure 4:**
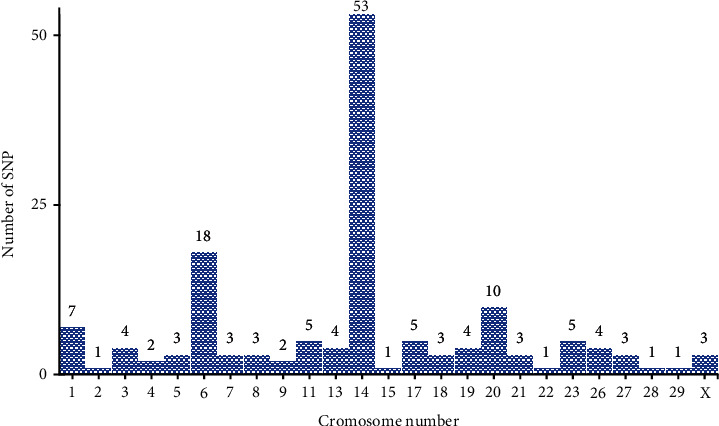
The number of SNPs significantly associated with multiple milk production traits in Holstein and its crossbreds.

A total of 136 SNPs were significantly associated with two or more milk production traits (MY, FY, PY, FP, and PP). According to Fontanesi et al. [22], rs109234250 was significantly associated with all milk production traits (MY, FY, PY, FP, and PP). As reported by [11, 12, 15, 17, 21, 22], 14 SNPs frequently affected four, 39 SNPs three, and 86 SNPs two of milk production traits. Number of significant SNPs associated with multiple milk production traits in Holstein and its crossbreds are shown in Figure 4. There was a greater number of SNPs frequently affected multiple milk production traits on chromosome 14. Thus, selection programs should focus on candidate genes and genomic regions that are known to influence multiple production traits.

Candidate genes, significant SNPs, and chromosomes that are simultaneously associated with more than one milk production trait are listed in Table 4. Several promising candidate genes were identified, including DGAT1, PLEC, Rho GTPase activating protein 39 (ARHGAP39), protein phosphatase 1 regulatory subunit 16A (PPP1R16A), and sphingomyelin phosphatase 5 (SMPD5). Genes retinol saturase (RETSAT), AarF domain containing kinase 5 (ADCK5), arc regulates transcription adhesion G protein-coupled receptor B1 (ARC-ADGRB1), Rho GTPase activating protein 39 (ARHGAP39), DGAT1, forkhead box H1 (FOXH1), PLEC, solute carrier family 52 member 2 (SLC52A2), and prolactin receptor (PRLR) frequently affected four milk production traits [11, 12, 15, 21].

## 4. Conclusion

This review summarized information on identified candidate genes and genomic regions associated with milk production traits in Holstein and its crossbreds from various regions of the world. Most of the identified SNPs and candidate genes were on chromosome 14. One of the challenges in dairy cattle selection is that milk production traits are expressed after the first calving. Candidate gene and genomic region information would permit earlier selection of males and females, shorten the generation interval, and accelerate genetic progress for milk production traits.

## Figures and Tables

**Table 1:** Candidate genes and genomic regions for MY in Holstein and its crossbreds.

SNP name	Gene	Chromosome	Breed	Number of cattle	Authors
rs41577598	BAIAP2	19	Canadian Holstein	462	[25]
rs41592943	GUCY2C	5			
rs41608371	FBLN5	21			
rs41632222	LOC512656	14			
rs41633664	LOC785291	1			
rs41643471	LOC508029	1			
rs41655901	GALNT6	5			
rs41656714	LOC407194	5			
rs41658330	FANCC	8			
rs43709850	LOC511740	3			
rs42517915	LOC788012	9	Chinese Holstein	2,093	[21]
rs43030751	LOC100139865	9			
rs41654691	INXA1	8	U.S. Holstein	1,654	[19]
rs42300745	TMX4	13			
rs42586116	PTBP2	3			
rs42586854	PTBP2	3			
rs42725189	PTBP2	3			
rs43408337	ULOC781500	4			
rs109289569	L00531776	14			
rs110384096	C-ICNG5	19			
rs110944623	PIGN	24			
rs41568120	PLCB1	13			
rs41643761	SLC25A2 I	21			
rs42914124	LOCI00140505	10			
rs109104203	INO80	10	Portuguese Holstein	526	[24]
rs109832473	CACNB2	13			
rs109942798	LOC525149	12			
rs110323635	MAPK15	14			
rs110476141	AQP4	24			
rs41580384	CACNB2	13			
rs41659095	GPAM	26			
rs43186715	LOC525149	12			
rs43272177	MARS	1			
rs41640548	ATP11B	1	Chinese Holstein	445	[18]
rs110281536	EEF2K	25			
rs41774442	GNA14	15	Thai Crossbreds	36	[8]
rs41776130	GNA14	15			
rs41776828	LRRC4C	15			
rs41779510	GNA14	15			
rs41796094	GNA14	8			
rs43705173	STAT1	2	Italian Holstein	45,115	[23]
rs41631082	ECI2	23	Russian Crossbreds	477	[9]
rs43364576	MACF1	3			
rs109295123	SPOPL, HNMT	2			
rs110748809	DTX1	17			
rs110425841	PODXL2	22	Colombian Holstein	150	[10]
rs110718748	ANKS1B	5			
rs41607880	TMEM229A	4			
rs41913085	VIT	11			
rs43483670	MAPK10	6			
rs110482506	GHR	20	US Holstein	294,079	[26]
rs110527224	GC	6			
rs137431035	PTGER4	20			
rs41938455	C6	20			
ss2019489562	UGDH	6			
rs41613423	CYP7B1	14	Chinese Holstein	295	[11]
rs474736745	PAIP1	20	French Holstein	6321	[13]
rs109355809	U6	14	Brazilian Crossbreds	337	[16]
rs109381761	SLC51A	1			
rs109704754	U6	18			
rs110565520	CD47	1			
rs41570140	FBXO11	11			
rs41642215	BET1	4			
rs41647684	BMPR1A	28			
rs42123132	SSBP2	7			
rs42215728	SLC24A2	8			
rs42271	SLC24A2	8			
rs42290054	CDH12	20			
rs42490796	EDIL3	7			
rs42583510	FBXO11	11			
rs42900126	ANXA5	6			
rs43424124	NUB1	4			
rs108973652	IREB2	21			
rs108977582	ITGB1BP1	11			
rs108982955	TMEM247	11			
rs109009656	MAP1B	20			
rs109099963	FAM135B	14			
rs109176086	KHDRBS3	14			
rs109327460	SLC35B3	23			
rs109840333	SLIT3	20			
rs109854193	FST	20			
rs109901641	FAM114A2	7			
rs109912809	U6	7			
rs109984167	HSPB3	20			
rs110192732	HES1	1			
rs29021936	ELAVL2	8			
rs41657410	PAM	7			
rs41919419	TANC2	19			
rs42040508	MBP	24			
rs42343030	GCNT2	23			
rs42615382	TEX35	16			
rs42674867	5S_rRNA	18			
rs42934321	LAMA3	24			
rs43419957	WDR86	4			
rs110535430	MARCHF10	19			
rs110632003	GRID2	6			
rs110775601	NPFFR2	6	Korean Holstein	2780	[17]
rs135477609	ADRA1B	7			
rs110527224	SLC4A4	6			
rs517703887	PKHD1	23			
rs524049037	GFRA2	8			
rs108962265	PITRM1	1	Chinese Holstein	999	[7]
rs110246034	PRMT6	3			

**Table 2:** Candidate genes and genomic regions for FY and FP in Holstein and its crossbreds.

SNP name	Gene	Chromosome	Trait	Breed	Number of cattle	Authors
rs29020642	LOC512171	1	FY	Canadian Holstein	462	[25]
rs41634488	LOC786403	1				
rs41637121	APP	1				
rs41588659	COL1A2	4				
rs41591894	ITPR2	5				
rs41592942	GUCY2C	5				
rs41652648	ITPR2	5				
rs41653025	LOC540856	10				
rs43703342	LOC514626	11				
rs41645253	MGC139244	24				
rs41569649	CAMK2G	28				
rs41653440	PSAP	28	FY			
rs41653491	LOC514949	28				
rs43709929	LOC514870	3				
rs29018853	LEC3	6				
rs41592660	LOC616136	9				
rs41657163	LOC535127	9				
rs43710950	TPM1	10				
rs41567322	TG	14				
rs41579063	BIG1	14				
rs41639879	LOC505156	17				
rs41641678	LOC514186	21				
rs41643783	MGC139789	21				
rs41648176	LOC515764	26				
rs29017368	LOC515967	5	FY	Chinese Holstein	2,093	[21]
rs41648982	LOC511240	5				
rs110090404	C14H8orf33	14				
rs41664719	LOC516454	X				
rs109948273	EIF2C2	1	FP			
rs41617243	KBTBD10	2				
rs41652649	ITPR2	5				
rs43499009	NFIB	8				
rs110704765	LOC526069	11				
rs110710474	GFI1B	11				
rs109436130	KHDRBS3	14				
rs109529219	RHPN1	14				
rs110143087	KCNK9	14				
rs110293317	KHDRBS3	14				
rs110323635	MAPK15	14				
rs110411273	GPR20	14				
rs110718625	KHDRBS3	14				
rs111022074	LOC100138440	14				
rs41567288	NIBP	14				
rs41576704	EIF2C2	14				
rs108995214	KHDRBS3	14				
rs109118650	LOC618755	14				
rs109225594	KCNK9	14				
rs109230014	KCNK9	14				
rs109241573	KHDRBS3	14				
rs109402117	LOC618755	14				
rs109476486	LYPD2	14				
rs109617015	ZC3H3	14				
rs109661298	EEF1D	14				
rs109670279	PTK2	14				
rs109742607	MAPK15	14				
rs110057993	KHDRBS3	14				
rs110165168	MIRN30D	14				
rs110339989	OPLAH	14				
rs110351374	COL22A1	14				
rs110351748	COL22A1	14				
rs110424520	GPR20	14				
rs110501942	LOC618755	14				
rs110502094	LOC100138440	14				
rs110522477	KHDRBS3	14				
rs110545496	KHDRBS3	14				
rs110626984	CYP11B1	14				
rs110775004	NIBP	14				
rs110892754	LOC524939	14				
rs111018678	NIBP	14				
rs41576704	EIF2C2	14				
rs41624797	PTK2	14				
rs41627764	ZNF623	14				
rs41630614	LOC785799	14				
rs41657812	LOC100138440	14				
rs42305942	LOC100138440	14				
rs42310935	LOC100138440	14				
rs55617160	NIBP	14				
rs109950724	LOC782348	5	FY	U.S. Holstein	1,654	[19]
rs110355546	ZBPI	13				
rs41639184	LPP	X				
rs110267314	LM03	5	FP			
rs41592948	GABARAPL1	5				
rs109146371	FOXHI	14				
rs109350371	LOC786966	14				
rs109421300	DGAT1	14				
rs109558046	VPS28	14				
rs109752439	ZAT34	14				
rs110017379	NIBP	14				
rs110060785	GPITIBP1	14				
rs110891564	KCNK9	14				
rs110091513	SART3	17				
rs109062793	AP1B4	X				
rs109116007	SYTL5	X				
rs41919985	FASN	19	FY	Italian Holstein	800	[22]
rs41613079	EPHA7	9	FP	Russian Holstein	61	[20]
rs42723319	EPHA7	9				
rs109350371	LOC786966	14				
rs109421300	DGAT1	14				
rs134390757	LxR-*α*	15	FY	Italian Holstein	45,115	[23]
rs135588030	ORL2	5	FP			
rs43349286	LPAAT	23				
rs41624917	PLCE1	26				
rs41670205	LRP1B	2	FP	Colombian Holstein	150	[10]
rs109245784	CLCN1	4				
rs43655765	SNRNP200	11				
rs110897514	PCDH18	17				
rs41571534	WSCD1	19				
rs41629750	GRINA	14	FP	Russian Crossbreds	477	[9]
rs109350371	PLEC	14				
rs109421300	DGAT1	14				
rs109968515	CYHR1	14				
rs110199901	ZNF696	14				
rs17870736	VPS28	14				
rs42406616	ABCC9	5	FY	US Holstein	294,079	[26]
rs42718234	ABCC9	5				
rs109350371	LOC786966	14				
rs133114040	EPS8	5	FP			
rs109146371	FOXH1	14	FP	Chinese Holstein	295	[11]
rs109752439	C14H8orf33	14				
rs208148726	AKT3	16				
rs43526055	ADRA1B	7	FY	Chinese Holstein	1220	[14]
rsl37676276	VIT	11				
rs135780687	GRPR	x				
rsl09528658	EF400	17				
rs42295213	EPHA6	1	FP			
rs136949224	SCARA5	8				
rs110825388	CPSF1/ADCK5	14	FY	French Holstein	6321	[13]
rs211210569	MGST1	5				
rs208248675	MGST1	5	FP			
rs136548039	HERC5	6				
rs109982707	PAEP	11				
rs109234250	DGAT1	14				
rs208675276	GPAT4 5′ UTR	27				
rs110199901	LY6H	14	FY	Chinese Holstein	300	[15]
rs109742607	MAPK15	14	FP			
rs41624797	PTK2	14				
Na rs	LY6H	14				
rs109476486	LYNX1	14				
rs109617015	VAMP2	14				
rs110339989	OPLAH	14				
rs137787931	MROH1	14				
rs17870736	VPS28	14				
rs41627764	MIR193A-2	14				
rs41630614	RPL8	14				
rs41596885	PDE4B	3	FY	Korean Holstein	2780	[17]
rs42314807	PDE4B	3				
rs43454033	ANO2	5				
rs109234250	DGAT1	14				
rs109326954	DGAT1	14				
rs109421300	DGAT1	14				
rs110812136	SPATC1	14				
rs135258919	HSF1	14				
rs208317364	DGAT1	14				
rs435871639	PKHD1	23				
rs109414214	EXT1	14	FP	Indian Crossbreds	96	[27]
rs109632163	NOV	14				
rs41614632	-	14				
rs41665025	SNTG1	14				
rs41730911	FABP, PMP2	14				
rs42485761	ZFPM2	14				
rs43067787	KCNB2	14				
rs108957364	AARD	14				
rs110390518	-	14				
rs110981268	SNX16	14				
rs81118743	RF00026	14				
rs43527533	TENM2	7	FY	Chinese Holstein	999	[7]
rs42206791	METTL15	15				
rs137260850	PLA2G4A	16				
rs109656599	CDH13	18				
rs109595510	RCSD1	3	FP			
rs210744919	MGST1	5				
rs133840542	SUPT2OH	12				
rs137071126	SLC52A2	14				
rs109278135	NOL4	24				
rs133996308	PLAU	28				

**Table 3:** Candidate genes and genomic regions for PY and PP in Holstein and its crossbreds.

SNP name	Gene	Chromosome	Trait	Breed	Number of cattle	Authors
rs41591535	LOC781748	4	PY	Canadian Holstein	462	[25]
rs29011990	MGC155155	8				
rs41636749	LOC538513	18				
rs41648723	CTBP2	26				
rs41606880	JDP1	28				
rs41650658	NRCAM	4	PP			
rs29014633	CACNG2	5				
rs41590827	RAC2	5				
rs41593881	HIF1A	10				
rs29021058	PLCG1	13				
rs41566192	MGC127374	13				
rs41637636	SLC38A3	22				
rs29016156	LOC517805	23				
rs109452554	ETS2	1	PY	Chinese Holstein	2,093	[21]
rs109680710	DIP2A	1				
rs41586699	PDE9A	1				
rs109271556	LOC781902	3				
rs109700191	SLC30A7	3				
rs29025951	LOC100138725	3				
rs41589462	LOC539739	3				
rs110896997	PDHA2	6				
rs41659807	LOC788115	9				
rs109819417	NKAIN3	14				
rs110618422	CSF2RB	5	PP			
rs42552739	NCF4	5				
rs81154068	HERC3	6				
rs29018333	LOC536367	6				
rs41622323	PKD2	6				
rs43463988	LOC100140991	6				
rs110805364	NIBP	14				
Na rs	GHR	20				
rs109181046	GDNF	20				
rs110679619	RICTOR	20				
rs29013890	LOC782833	20				
rs29014437	LOC782284	20				
rs29018751	NIPBL	20				
rs41574319	RAI14	20				
rs41581059	LOC100138964	20				
rs41937533	LOC518808	20				
rs41941633	FYB	20				
rs41941646	C9	20				
rs41942492	NIPBL	20				
rs41945918	LOC782462	20				
rs42954630	NIPBL	20				
rs109583255	CRABP1	21	PY	U.S. Holstein	1,654	[19]
rs29011 699	ALDH5A1	23				
rs110736402	L424P2	X				
rs41598282	ATP1B4	X				
rs110675489	SREBF2	5	PP			
rs110931400	PDGFRA	6				
rs111032162	PDGFRA	6				
rs109024105	RPL37	9				
rs109352200	USP 3 8	17				
rs41256775	L0052 8054	17				
rs109375227	Artora	18				
rs109570377	CX036	X				
rs108964624	LOC781178	X				
rs110304690	COL4	X				
rs110328561	LOC781178	X				
rs41628209	LOC616260	X				
rs42967999	LOC781902	3				
rs41569048	PTHLH	5	PP	Dutch Holstein	1,713	[28]
rs41640170	HEATR7B2	20				
rs42529901	LOC788223	6	PY	Portuguese Holstein	526	[24]
rs42749054	MAPK9	7				
rs109593545	BRD7	18				
rs41255191	OTUD7A	21				
rs109826203	ACSM5	25				
Na rs	CSN3	6	PP	Italian Holstein	800	[22]
rs109163366	PPARGC1A	6				
rs110980619	HPS3	1	PY	Russian Holstein	61	[20]
rs41647284	SLC16A7	5				
rs41601570	ARL15	20	PP			
rs109007595	POU1F2	1	PP	Italian Holstein	45,115	[23]
No rs	PPARGC1A	6				
rs109579682	PPARGC1A	6				
rs43703011	CSN3	6				
rs43703017	CSN5	6				
rs137457402	LPIN1	11				
rs41255713	CCL3	19				
rs109136815	GHR	20				
rs29023352	INSIG1	4	PP	Colombian Holstein	150	[10]
rs41624303	Arntl2	5				
rs29014693	NMBR	9				
rs41772701	CAPN5	15				
rs110425841	PODXL2	22				
rs41576177	OSBPL1A	24				
rs109670279	BOD1L1	6	PP	Russian Crossbreds	477	[9]
rs43592374	UBE3D	9				
rs43710185	IL15	17				
rs109774038	NDUFA9	5	PY	US Holstein	294,079	[26]
rs132896414	GALNT8	5				
rs379188781	CCND2	5				
rs135228504	ZNF34	14				
rs109558046	VPS13B	14	PP			
rs110478571	NLK	19	PP	Chinese Holstein	769	[12]
rs109558046	LOC104975266	20				
rs110000229	GHR	20				
rs41257416	HCN1	20				
rs42418694	SND1	4	PY	Chinese Holstein	295	[11]
rs41579063	ARFGEF1	14				
rs41629750	SPATC1	1	PP			
rs109748124	TNFSF10	1				
rs41602511	YTHDF3	1				
rs41617243	KLHL41	2				
rs110256520	ATP1A1	3				
rs110420888	ATP1A1	3				
rs134541510	RPAP3	5				
rs137408198	CD27	5				
rs29015155	RPAP3	5				
rs41592948	GABARAPL1	5				
rs110672723	CSN2	6				
rs110727998	GML	14				
rs41579932	ARMC1	14				
rs110834172	KSR2	17				
rs29014438	SLC1A3	20				
rs29021190	SLC1A3	20				
rs41639260	GHR	20				
rs41934711	CCNB1	20				
rs134480235	SLCO1A2	5	PY	Chinese Holstein	1220	[14]
rs109875012	ZNF384	5	PP			
rs108996837	EXOC3L4	21				
rs136903701	ABCC9	5	PY	French Holstein	6321	[13]
rs43703011	CSN2	6				
rs134511693	EFNA4	3	PP			
rs456403270	TBC1D22A	5				
rs383909572	HSTN	6				
rs41622323	PKD2	6				
rs135458711	SLC39A4	14				
rs110144962	APOA4	15				
rs208817293	WDR74/U2	29				
rs378017490	PICALM	29				
rs799074643	UMPS	1	PY	Korean Holstein	2780	[17]
rs211419403	GFRA2	8				
rs109957491	MFSD1	1	PY	Chinese Holstein	999	[7]
rs109097262	PLCB4	13				
rs41906111	MY01D	19				
rs43496186	WNT9A	7	PP			
rs109425744	CORO2B	10				
rs135708753	ATP11A	12				
rs132711282	FBX O 43	14				
rs43002440	KHDRBS3	14				
rs110387086	MLXIP	17				

**Table 4:** Candidate genes and genomic regions affecting multiple milk production traits in Holstein and its crossbreds.

SNP name	Gene	Chromosome	Traits	Breed	Authors
rs41629125	ITGB5	1	MY, FY, and PY	Canadian Holstein	[25]
rs41631818	MGC128242	1	MY, FY, and PY		
rs41587408	PDZK1	3	FP and PP		
rs41578761	LOC529633	7	FY and PY		
rs41662488	LOC785678	9	MY and PY		
rs41569023	ROCK2	11	MY nad PY		
rs41579049	5-OPase	14	FY and FP		
rs41580517	KCNQ3	14	FY and FP		
rs41587081	ZFHX4	14	MY and FP		
rs41628862	BIG1	14	MY and PY		
rs41633631	TG	14	MY and FP		
rs41570561	SCARB1	17	FP and PP		
rs41581694	FOXA3	18	MY and PY		
rs41585246	SERPINA3-5	21	MY and PY		
rs41644615	SERPINA5	21	MY, FY, and PY		
rs41640789	POLR1C	23	FY and FP		
rs41643632	LOC534225	23	MY and PY		
rs43282015	LOC614166	1	MY and PY	Chinese Holstein	[21]
rs41663626	LOC534011	3	MY and PY		
No rs	HAL	5	MY and PY		
rs110727998	GML	14	MY and FP		
No rs	COL22A1	14	MY and PY		
rs109146371	FOXH1	14	MY, FY, and PP		
rs109350371	LOC786966	14	MY, FY, PY, and PP		
rs109421300	DGAT1	14	MY, FY, and PP		
rs109752439	C14H8orf33	14	MY and PY		
rs109968515	CYHR1	14	MY, FY, and PP		
rs110017379	NIBP	14	MY, PY, and FP		
rs110060785	GPIHBP1	14	MY, PY, and FP		
rs110199901	ZNF66	14	MY and FP		
rs110622450	COL22A1	14	MY and FP		
rs17870736	VPS28	14	MY, FY, and PP		
rs41256919	MAF1	14	MY and FP		
rs41629750	GRINA	14	MY, FP, and PP		
rs41583200	C26H10orf84	26	MY and PY		
rs42462826	FKBP2	1	FY and PY	U.S. Holstein	[19]
rs109250591	ITC14	1	FY and PY		
rs109703572		9	MY and FY		
rs43101493	GNAS	13	MY, FY, and PY		
rs41585412	GNAS	13	MY, FY, and PY		
rs41630667	GNAS	13	MY, FY, and PY		
rs111018678	NIBP	14	FP and PP		
rs42422883	HHIP	17	FY and PY		
rs108993234	PGLYRPI	18	MY, FY, and PY		
rs29010796	MAF	18	FY and PY		
rs109343058	GPRI 10	23	FY and PY		
rs110886345		26	FY, PY, and PP		
rs110898125		26	FP and PP		
rs41626960	MGMT	26	FY and PY		
rs42227052	FUT10	27	MY, FY, and PY		
rs41575183		27	FY and PY		
rs109335394		X	FY and PY		
rs41579345	GLRA2	X	FY and PY		
rs41628209	LOC616260	X	FY and PY		
rs41255709	CXCR1	2	MY, FY, and PY	Italian Holstein	[22]
rs29004485	LEP	4	MY, FY, and PY		
rs29004488	LEP	4	MY, FY, and PY		
rs109299401	CSN3	6	PY and PP		
rs133669403	PPARGC1A	6	MY and PY		
rs43703011	CSN2	6	MY and PY		
rs109625649	LGB	11	MY and PY		
rs110066229	LGB	11	MY and PY		
rs41255679	LGB	11	MY and PY		
No rs	CRH	14	PY and PP		
rs109162116	DGAT1	14	MY, FY, PY, and FP		
rs109234250	DGAT1	14	MY, FY, PY, FP, and PP		
rs41580467	TG	14	MY and FP		
rs41758918	TPH1	15	MY and PY		
rs42321611	PRLR	20	MY, FY, PY, and PP		
rs42714482	THRSP	29	MY, FY, and PY		
rs29017970	LOC104973750	13	MY and PY	Russian Holstein	[20]
rs41850250	TRAFD1	17	MY and PY		
rs135514413	ETS2	1	FP and PP	Italian Holstein	[23]
rs41608610	DGKG	1	MY, FP, and PP		
rs109299401	CSN2	6	PY and PP		
rs110981354	CSN1S1	6	FY, FP, and PP		
rs133669403	PPARGC1A	6	MY, FP, and PP		
rs43703013	CSN4	6	MY and PP		
rs43703015	CSN4	6	MY, FY, and PY		
rs43706475	CSN3	6	MY and PY		
rs110590698	LPL	8	FY, FP, and PP		
rs8193666	TLR4	8	MY, FP, and PP		
rs110757796	FABP4	14	MY and PY		
rs110937773	FGF2	17	MY and FY		
rs109578101	STAT5A	19	MY, PY, and FP		
rs109686238	CCL3	19	FY and PY		
rs109428015	PRLR	20	FP and PP		
rs41923484	GHR	20	PY, FP, and PP		
rs41257077	PI	21	FY and PY		
rs43765462	LTF	22	FY and FP		
rs43706495	BTN1A1	23	MY and FP		
rs109913786	AGPAT6	27	FY and FP		
rs381714237	FCGR2B	3	MY, PY, and PP	US Holstein	[26]
rs110825637	MGST1	5	FY and FP	Chinese Holstein	[12]
rs137735153	PLEKHA5	5	FY and FP		
rs109901151	SLC4A4	6	MY and PY		
rs110694875	ADAMTS3	6	MY and PY		
rs136639319	TBC1D1	6	FP and PP		
rs137147462	GC	6	MY and PY		
rs378415122	CENPE	6	MY, FY, and PY		
rs385060942	CENPE	6	MY, FY, and PY		
rs453960300	CENPE	6	MY, FY, and PY		
ss2137349051	CENPE	6	MY, FY, and PY		
ss2137349053	CENPE	6	MY, FY, and PY		
rs377943075	ACSBG2	7	FY and PP		
rs134985825	RETSAT	11	MY, FY, PY, and PP		
rs109146371	PPP1R16A	14	MY, FY, PY, and PP		
rs109350371	PLEC	14	MY, FY, PY, and PP		
rs109558046	ARC-ADGRB1	14	MY, FY, PY, and FP		
rs110914335	LY6H (d)	14	MY and PY		
rs134444531	NLK	19	PY and PP		
rs41573457	MRPS30	20	MY and PP		
ss2137349058	MAP3K1	20	MY, FY, and PY		
rs41589462	KCND3	3	FP and PP	Chinese Holstein	[11]
rs136195618	PROP1	7	MY and PY		
rs109350371	PLEC	14	MY, PY, FP, and PP		
rs109421300	DGAT1	14	MY, FP, and PP		
rs109968515	CYHR1	14	FY and PY		
rs110017379	TRAPPC9	14	MY, FP, and PP		
rs41579243	FAM135B	14	FP and PP		
rs109646517	MTMR3	17	MY and PY		
rs29014437	SLC1A3	20	MY and PP		
rs41580312	OSMR	20	MY and PP		
rs41942492	NIPBL	20	FP and PP		
rs42340412	ARID5B	28	FP and PP		
rs109421300	DGAT1	14	FP and PP	Chinese Holstein	[14]
rs109007040	VPS13B	14	MY, FP, and PP	French Holstein	[13]
rs109421300	DGAT1	14	MY and PY		
rs41921161	CCDC57	19	FY and FP		
rs110231369	ARHGEF28	20	FY and PY		
rs385640152	GHR	20	FP and PP		
No rs	ADCK5	14	MY, FY, FP, and PP	Chinese Holstein	[15]
No rs	FOXH1	14	MY, FY, FP, and PP		
No rs	GRINA	14	FY, FP, and PP		
No rs	SLC52A2	14	MY, FY, FP, and PP		
rs109146371	FOXH1	14	MY, FY, FP, and PP		
rs109752439	C14H8orf33	14	FY and FP		
rs110323635	MAPK15	14	FY and FP		
rs721532493	PALLD	8	MY, FY, and PY	Korean Holsteins	[17]
rs109421300	DGAT1	14	MY, FY, PY, and PP		
rs135549651	SMPD5	14	MY, FY, PY, and PP		
rs207655744	HSF1	14	MY and FY		
rs208640292	HSF1	14	MY and FY		
rs209876151	DGAT1	14	MY and FY		
rs211016627	HSF1	14	MY and FY		
rs211223469	DGAT1	14	MY and FY		
rs211282745	HSF1	14	MY and FY		
rs384957047	DGAT1	14	MY and FY		
rs380223715	PKHD1	23	MY and PY		
